# Indents

**Published:** 1905-06-15

**Authors:** 


### INDENTS.

**☞ Brief Articles of Technical or Scientific Value will receive notice and comment. Contributions invited.**

**Antiseptic Root Filling.** After the canals have been sterilized, flush them with a solution of campho-phenique in which thymol has been dissolved in the proportion of forty grains of thymol to the ounce of campho-phenique. Then take a canal plugger and attach to it, after warming the large end of a gutta-percha point. Dip the point into the campho-phenique and thymol solution and while still wet dip it into dry iodoform and insert into the canal and force to the end of the root. In my judgment no antiseptic has ever been used so successfully in canal treatments as iodoform. It seems to neutralize the poisonous consequences of bacteria.—J. Leon Williams, in *Cosmos*.

**Impression of Cavity.** In case of a difficult cavity, large and complicated and running under the gingival margin, first get a fairly accurate impression with pink base-plate gutta-percha. Push it well to place and cool with ice water or chloride of ethyl spray. Remove and trim; then paint the cavity with vaseline, mix cement to medium consistency, place it on the impression and push to place in the cavity. Scrape off excess of cement and when set remove and invest in plaster, letting it stand up well above the impression making a depression into which pack amalgam. When set break away the plaster, warm the gutta-percha slightly and remove it, and place the amalgam mass with cement attached in ammonia-water over night. The cement will dissolve away with no danger of injuring the firm lines and frail corners of the amalgam die. With this accurate duplicate of the cavity you gain time in making your swaged matrix.—W. H. Taggart, *Dental Digest*.

**Mummifying Pulp Remnants.** R. M. Ratliff, in the May *Brief*, relates that he has used the mummifying paste for four years in over five hundred cases, with only four known failures, and gives his method as follows: Apply to the exposed pulp a nerve paste made of equal parts of arsenic and cocaine to which enough creosote is added to make the paste of desirable consistence. This is sealed in the cavity and the patient instructed to return in twenty-four hours. The pulp is then found insensible. The bulbous part of the pulp is removed and as much of that in the root canals as can be reached after reaming them out a little over half their length. The mummifying paste is then inserted and forced into the canals with a pledget of cotton and a burnisher and the filling completed.

**Cementing Surface of Inlays.** Treating the surface of a porcelain inlay, so that there will be a chemical continuity of structure when cement is applied. It has been found that zinc oxid, as available in the usual cement powder, can be so fused to a surface of porcelain that cement applied thereto will literally unite with and become continuous with the particles of zinc oxid presented. This can be accomplished for retention of an inlay, by painting into the matrix—keeping back of the edge, of course—a mixture of two parts of cement powder and three parts of the porcelain body about to be used, the merest film being the proper quantity. If the matrix be then filled with the plain porcelain and the inlay fused and completed as usual there will be presented, on stripping off the matrix, a glazed surface which can be easily broken, exposing a thin porous layer which not only gives mechanical retention similar to an etched surface, but presents zinc oxid particles with which the cement will become united.

**Barred Out.** There is no hope of ever getting into the charmed social circle, or of rising therein, for the man who cleans other people, or any part of them—even their teeth. To clean people is to remove their dirt, and to remove their dirt as such and by its real name is to be forever barred from that which to some of the frail-minded of the profession is quite as desirable as life itself. People should be made to clean themselves dentally, after which the way is open to the professional man to remove calcarceous deposits, deep-seated stains, etc., which from the nature of the case is beyond the mere cleansing capabilities of the patient and his limited and inefficient toilet outfit. This is subjecting the teeth to prophylactic treatment! It may be "cleaning" to the public; but to the professional man that word should be unknown, and never used to describe his part of the renovating operation. "What's the difference?" asks some one; "It's only in the name." True; but that's just the difference between a service that suggests the menial and one that can be rendered only by one possessing knowledge and skill beyond the mere application of soap and water. What dentistry needs in this matter is a new word and several derivatives therefrom, that will rid it for all time of such words as "clean" and "cleaning." to describe the professional part of the operation in question. Fellow feeling alone for the socially ambitious should prompt the word-mongers of the profession to give us the terms of which we stand in such sore need.—*Office and Laboratory.*

**A Reliable Means of Producing Local Anesthesia.**

W. Clyde Davis, B. S., M.D., D.D.S., claims the following advantages for his painless method of pulp-extirpation: Absence of pain during the entire procedure; control of hemorrhage; a dry root canal for subsequent filling; less percentage of subquent periodontitis; color of tooth always preserved; the last two being advantages over the arsenical method.

The author depends upon adrenalin with cocain to produce the necessary degree of anesthesia in this method, as well as in tooth extraction. He suggests the following mixture: In a small dish, as a salt cup, place one or two drops of water, and then add one-sixth grain compressed tablet of cocain. Crush and stir until you get a clear solution. Then add four to six drops of adrenalin chloride solution, to this add witch hazel, sufficient to fill hypodermic syringe. Inject as any other local anesthetic is used.

At Dr. Davis' suggestion, Messrs. Parke, Davis & Co. have brought out a hypodermic tablet of adrenalin and cocain which is especially intended for the extemporaneous preparation of anesthetic solutions. Each tablet contains adrenalin, 1.300 grain; cocain hydro-chlorate, one-sixth grain. Don't make injections of any sort in close proximity to pus collections, owing to the risk of causing extravasation of pyogenic bacteria with troublesome sequelæ. The judicious use of this solution is attended with less danger and subsequent soreness, while the anesthesia is more profound than with any method previously used.

**Dental Laws.** Nebraska's new dental law compels graduates to be examined for registration and the fee is $25.00. The law also contains a reciprocity clause.—New Mexico also has a new law. Candidates for examination must present either a piploma or a certificate signed by three reputable legalized practitioners of the State. Every licensed practitioner must pay three dollars each year to keep his license intact.—Vermont's new law permits every one to extract teeth whether they have ever studied dentistry or not.—Ellsworth Glenn, who was fined $50.00 in Columbus, Ohio, for practicing without a license, has appealed his case to the supreme court.—George L. Terry, who owned an advertising dental office in New Jersey, was acquitted of violating the State laws by a jury. The defense being that he was only the proprietor and did none of the operative work, which was done by an assistant who was legally qualified.—A proposed amendment to the Rhode Island dental law makes it compulsory for the State Examining Board to render to every candidate to whom it declines to give a license to practice a statement of the reasons therefor. The defeated candidate may then appeal his case to the supreme court if he pleases, providing it is done in ten days after receiving the notice.—That portion of the Washington State law which requires that any person owning or managing a dental office shall procure a license to practice, has been declared unconstitutional by the State supreme court, on the ground that the owning or managing of a dental office is not inimical to the public health, and that no personal or property rights are threatened thereby.—The Governor of Pennsylvania has vetoed the revised dental law of that State just passed by the Legislature. His reasons are that the bill makes a violation of the law punishable by a fine and also imprisonment. He does not believe the State should imprison unlicensed practitioners. He also objects to making appointments of examiners from nominees of the State Dental Society.

**Open-face Crowns.** Unquestionably the objections urged against open-face crowns are well founded. At their best they have elements of weakness difficult to overcome. Nevertheless, properly made and properly placed, they have a field of usefulness all their own. Upon bicuspid teeth I have found them satisfactory and durable, especially so in cases which admit of the open face being entirely open. When the bar connecting the two sides at the gum margin can be dispensed with, the sides can be made to spring apart as the crown passes over the tooth, and to embrace more closely the tooth-neck when it is in place. The peculiar shape of the cuspid and incisor teeth not only increases the difficulty of making the crown fit accurately, but it also hampers us when cementing it in place. We miss the piston-like action of the tooth which assists so much in forcing the cement solidly into the intervening space when a full crown is pressed into place. Not only does the cement escape through the open face, but the screw-like motion necessary when manipulating it into place so displaces the cement that a thorough and compact filling of the space between the tooth and the crown becomes impossible. As a natural result the cementing is imperfect. To so shape the tooth as to avoid this would, in many cases, result in so serious a mutilation that devitalization and excision might be preferable.

In a recent English dental journal a suggestion is made that perhaps may have practical value. The writer suggests instead of mutilating the tooth, to build it up at the neck by first fitting to each side of the neck of the tooth a piece of thin gold plate; to hold this in place one or two small holes are drilled through the plate and extended a short distance into the tooth into which pins are fitted and soldered to the plate. Plate and solder are now added in sufficient amount to give the tooth a desirable shape and filed to conform to the contour of the tooth. These are polished and cemented in place. When the finished crown extending over these additions is in position, there is no risk of their being displaced. Carefully carried out, this expedient seems promising, and is well worth a trial.—Dr. Wm. H. Trueman, *Brief*.

**Ancient and Modern Dentistry.**

We extract the following interesting statements from the address of the *Regierungsrath*, of Basel, Switzerland, who welcomed the meeting of the recent International Dental Congress which met in Basel.

"Our interest is all the greater because we have the honor to see in our midst the leading representatives of a profession which in a comparatively short time has achieved triumphs of accomplishment and which is still hurrying from success to success. To prove this I need not go back to remote centuries, when the art or, at those times, rather the martyrdom of extracting teeth had been adopted by King John to make a Jewish merchant at Bristol, after ten extractions, hand over to the crown ten thousand marks of silver. Nor will I depict those barber-surgeries of our German towns, where, under the strict rules of a guild, dental operations were performed on the unlucky victims. Basel, too, was blessed with a great number of such demi-medical torture chambers, which vegetated under the superintendence of an honorary gild, called the 'Golden Star.' Tooth-pulling charlatans also abounded at fairs and other festivals, accompanied by infernal music, to oversound the howling of the patients. Dentists, as depicted by Dutch painters, especially by Teniers in his renowned Kasseler painting, are not without intimate charm, but the modern observer, in spite of all the admiration for the artist's talent, cannot avoid feeling a certain satisfaction when comparing those shops of the seventeenth with the modern dental offices.

"We need not ramble so very far afield if we wish to contemplate the glorious development of the dental science, for in Basel it is fully illustrated. The remembrances of my youth reach back as far as the latter part of 1850-1860, and the call upon a dentist then is still one of the most vivid impressions. How simple was in those years such a room, and how simple—to express myself mildly—was the treatment. And now, how changed, what art, what a carefulness, what a study and what a success! To whom do we owe all this? I believe chiefly to those energetic men of Anglo-Saxon blood, who, on the other side of the Channel and on the other side of the ocean, have worked indefatigably in the field of dentistry and who refused to be satisfied except with the best. Many of them have come over to us, and our young men, desirous of knowledge, have gone over to them and through them acquired those accomplishments in dental art and science which at home became a blessing for so many a suffering fellow-citizen."—*Dental Brief.*

**Southern Dental Association.** In the report of the meeting of the Southern Branch of the National Dental Association in the *Dental Brief* for April, is this assertion: "Some doubt has been felt recently regarding the loyalty of the Southern dentists to the Southern branch *and the parent body, the National Dental Association.*" (The italics are ours.) Knowing, as we well do, the minds of Southern dentists, the italicised part of the assertion is uncalled for by any direct or indirect evidence. There has never been any disaffection of the dentists of the South to the National Dental Association. Never has been, nor ever will be, as the supreme national representative body; but there are numbers of Southern dentists who object to the branch feature of this national body, as a substitute for a distinctive Southern sectional dental association. Sectional, not in a political or hostile sense, but simply as a gathering together of the profession of a certain territory of the Nation, with a big N, if it pleases some folks to have it put that way. Is it treason to the National Association or to the United States for the dentists of the Southern States to assemble in convention independently of any other organization? We trow not. Then let us alone. In our opinion there should be no branch either Northern, Western or Southern to the National Association. Why should there be? Is it necessary for the life of the National body that a branch—especially a Southern branch—should be annexed to it? Is it essential to the success of a sectional dental association, most particularly a Southern dental association, that it should be attached to the National body? Is it really detrimental to the interests and welfare of the National body that a sectional body should exist and thrive?

We do not believe that the National body would suffer baneful effects from the existence of sectional societies. It has not from the independent associations in the North and West and we are sure it would not from one in the South. This is why we have advocated and aided the organization of the Chapin Harris Dental Society, a sectional dental Southern association. We are free though to say that if it pleases the members of the Southern branch of the National to continue its existence, they certainly are at liberty to do so. We would not advise antagonism between it and the new society. Both can prosper and do much good independent of each other. But let there be an end to the impression that we at the South, who favor an independent association, are unfriendly and treacherous to the National association, for it is not true. The merging of the two old associations to form the National did not bind the different parts of the Union in a compact not to form independent bodies.—*Dental Hints*.

**Practical Things That Count.** Operating tables may be kept in a neat, presentable condition by using blotting paper covers. Such a cover will absorb all moisture from instruments and cotton pellets and prevent the spreading of drops of medicines. They may be readily changed when soiled and are cheap. The bracket table should be provided with a receptacle for waste cotton pellets and dressings instead of allowing them to be strewed around the chair and floor. A good receptacle can be made from a colored glass pomade jar into the top of which a star is cut. The points of the star will engage the cotton and clean the points of the pliers quickly. The jar may be scalded out and kept clean and free from odor.

The use of clean operating table covers and cotton receptacles will eliminate a great deal of the disagreeable odor so common to dental offices.

A small piece of pure soap will be found useful in many ways during operations. The mouth mirror may be kept from clouding by coating the glass with dry soap, and then wiping clean with a dry napkin. The edge of a sandpaper disk will not catch in the rubber dam, when polishing fillings, if it is first run in the soap. Disks and strips will cut faster and polish better if slightly soaked, on the grit side, before using. Disks and strips thus prepared, that are used for polishing gold fillings, will retain the particles of gold, and if saved and refined will more than pay for the trouble. Rubber dam will slip easily over the teeth if soaked around the holes. Ligature silk when soaked slips easily between the teeth. Use pure soap and sparingly and it will not be disagreeable to the patient.

The excising of a natural crown of a tooth, when a dowel crown is to be used, may be done with a square reel cross-cut enamel fissure bur, gauge No. 1, cutting through the tooth at right angles to its long axis and following closely the gingival border of the gum. By this method little grinding of the root is required and laceration of the gums is avoided.

A Richmond or any banded dowel crown may be removed by slitting the band and drilling between the root and cap with a square drill until the pin is reached and passed a little to one side. The pin may then be cut with a cylindrical cross-cut bur. This will allow the ready removal of the cap and give direct access to the root for the removal of the balance of the pin.

When impressions are to be taken, note all conditions of the mouth carefully. If a thick, viscous saliva be present it may be overcome by rinsing the mouth thoroughly with milk of magnesia. The adhesion of the impression material to the teeth may be prevented by coating them slightly with vaselin or by rinsing the mouth with milk of magnesia just before the operation.

When working with wax in the laboratory, use a large common school slate for a bench cover; it will catch all pieces and drops of melted wax and when removed leaves the bench clean and ready for the next work. Wax spots on a bench may be very annoying when gold work is being done.

A small brush wheel with a single row of moderately stiff bristles is excellent for polishing around plain teeth in vulcanite work. If wet soap is applied to the bristles they will retain the wet pumice and cut like a knife. Soap rubbed on a felt buff wheel will retain the wet pumice, causing it to cut much faster and considerably lessening the time and labor of polishing an artificial denture.

Plaster models of orthodontia cases, etc., may be hardened and given a marble-like surface by boiling them in stearin.

The models must be thoroughly dry before putting them into the boiling stearin. Use a double boiler, like a glue pot, for melting the stearin.

The light of the operating chair may be very much improved in the late afternoon or on dark, hazy days, by using white linen curtains as reflectors. The principal one is an ordinary roller shade fastened at the top of the window casing. The curtain to be the full width of the window and of sufficient length to allow of its being drawn out over the operating chair, above the operator's head, and attached to some fixed point in the wall or screen. The spring in the curtain fixture, if left uncaught, will be sufficient to keep the curtain taut. This curtain, with its glazed white surface sloping downward and backward from the top of the window opening, will direct the rays of light on to the operating field instead of permitting them to be dispersed into the room. The system of curtains may be carried much further to good advantage. Broad white curtains may be attached to brackets swinging at right angles to the window frame and adjusted at an angle that will direct the light rays as desired. By this means the operating chair may be easily and quickly inclosed in a box having white reflecting sides. The dead white of the curtains seems to soften the intensity of the light so that it is not unpleasant for the patient and causes no particular eye strain on the operator. The use of curtain reflectors has enabled me many times to continue operations without the aid of an artificial light which is such a terrific strain on the eyes of both operator and patient.—Dr. F. C. Brush, in *Dental Brief*.

